# The true costs of participatory sanitation: Evidence from community-led total sanitation studies in Ghana and Ethiopia

**DOI:** 10.1016/j.scitotenv.2017.05.279

**Published:** 2017-12-01

**Authors:** Jonny Crocker, Darren Saywell, Katherine F. Shields, Pete Kolsky, Jamie Bartram

**Affiliations:** a The Water Institute, University of North Carolina at Chapel Hill, 148 Rosenau Hall, CB #7431, Chapel Hill, NC 27599-7431, USA; b Plan International USA, 1255 23rd Swt NW Suite 300, Washington, DC 20037, USA

**Keywords:** Cost, Process, Behavior-change, Sanitation, Hygiene, CLTS

## Abstract

Evidence on sanitation and hygiene program costs is used for many purposes. The few studies that report costs use top-down costing methods that are inaccurate and inappropriate. Community-led total sanitation (CLTS) is a participatory behavior-change approach that presents difficulties for cost analysis. We used implementation tracking and bottom-up, activity-based costing to assess the process, program costs, and local investments for four CLTS interventions in Ghana and Ethiopia. Data collection included implementation checklists, surveys, and financial records review. Financial costs and value-of-time spent on CLTS by different actors were assessed. Results are disaggregated by intervention, cost category, actor, geographic area, and project month. The average household size was 4.0 people in Ghana, and 5.8 people in Ethiopia. The program cost of CLTS was $30.34–$81.56 per household targeted in Ghana, and $14.15–$19.21 in Ethiopia. Most program costs were from training for three of four interventions. Local investments ranged from $7.93–$22.36 per household targeted in Ghana, and $2.35–$3.41 in Ethiopia. This is the first study to present comprehensive, disaggregated costs of a sanitation and hygiene behavior-change intervention. The findings can be used to inform policy and finance decisions, plan program scale-up, perform cost-effectiveness and benefit studies, and compare different interventions. The costing method is applicable to other public health behavior-change programs.

## Highlights

• Bottom-up cost analysis of community-led total sanitation in Ghana and Ethiopia
• The program cost was $30–82 per household targeted in Ghana, and $14–19 in Ethiopia.
• Local investments were $8–22 per household targeted in Ghana, and $2–3 in Ethiopia.
• The findings are relevant for policy, practice, and further research.

## Graphical abstract

**Figure:**
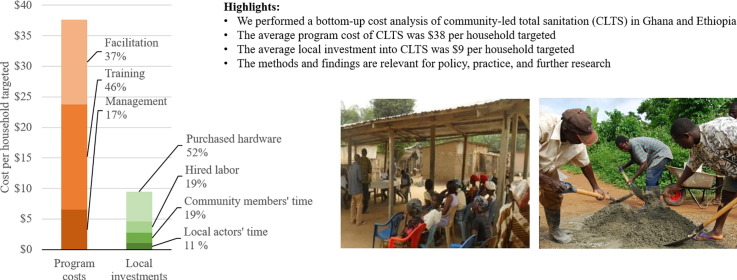

## 1 Introduction

Cost evidence informs policies, program design and scale-up, and research. Such evidence is lacking for water, sanitation, and hygiene (WaSH) programs that are participatory, involve capacity building, or target behavior-change. Improving this evidence is a priority, as meeting the Sustainable Development Goals (SDGs) will necessitate both a scale-up of efforts to meet universal targets, and a shift in the means of implementation toward capacity building, local participation, and behaviors (UN General Assembly, 2015).

WaSH and other public health programs have characteristics that make costing difficult: complex institutional arrangements, cross-subsidies, flexible implementation, and local investments. Complex institutional arrangements spread costs across organizations, resulting in inconsistent and incomplete financial tracking. Cross-subsidies arise when programs share resources (such as vehicles or training). Participatory, behavior-change programs are inherently flexible, extensively adapted, and field activities often do not match workplans or budgets. Local actors and communities contributing time or money (“local investments”) is common in participatory behavior-change programs.

Community-led total sanitation (CLTS) epitomizes these costing challenges. CLTS is a participatory approach in which facilitators visit villages and trigger awareness of sanitation issues during a community meeting. Facilitators then perform follow-up visits to villages to generate a community-wide effort to become open defecation free (ODF). Eliminating open defecation is included in the SDGs (UN General Assembly, 2015), as open defecation can cause malnutrition (Dangour et al., 2013), child stunting (Spears, 2013), and death (Prüss-Ustün et al., 2014). CLTS has spread to over 60 countries since 2000, in part because of perceived low cost: it rarely includes subsidized latrines (Institute of Development Studies, 2016, Kar and Chambers, 2008).

Costing methods are either top-down or bottom-up. Top-down costing (TDC) involves dividing a program's budget or total expenditures by the number of units (villages, households, individuals) targeted or reached. It is appealing due to its use of minimal, routinely collected data (budgets, expenditures, population targeted or reached), simple analysis, and that it can be retrospective. TDC can be accurate when: budget and expenditures represent *all* program costs, and *only* that program's costs, and the population served is unambiguous; conditions uncommon for WaSH programs. When cross-subsidies exist, TDC will under- or over-estimate costs depending on which program purchased shared resources. Neglecting local investments leads to underestimated total costs, leaves potentially disadvantaged beneficiaries out of cost considerations, and contributes to poorly informed policy (Garber and Phelps, 1997). In the absence of these conditions, one advantage of TDC is that it can capture management and overhead costs better than bottom-up costing (BUC). TDC does not allow disaggregation of costs by category (e.g. management, training, hardware), actor (e.g. government, non-governmental organization, community), time (e.g. by month), or by project or setting (Chapko et al., 2009). It is inappropriate when these factors are of interest, as is frequent in WaSH.

BUC involves careful tracking and analysis of implementation to calculate costs and assign them to activities. Regular program activities (timesheets, household surveys) can be adapted to collect the data needed for BUC. However, many cost analyses are done retrospectively, which precludes BUC. Additionally, the analysis is time consuming, complex, and expensive (Carey and Burgess, 2000), which could explain BUC's scarcity. BUC is more appropriate than TDC for the complexity of WaSH. BUC overcomes the main sources of error and bias (Adam et al., 2003), and enables analysis of variation in cost, economies of scale, and comparison of interventions (Chapko et al., 2009), which are valuable for program design and management.

While there is a growing body of evidence on the effectiveness of sanitation interventions (Garn et al., 2017), cost evidence is lacking. Several authors have compiled secondary data to model the costs and benefits of achieving global WaSH targets (Haller et al., 2007, Hutton and Varughese, 2016), or to compare different interventions (Hutton et al., 2007, Whittington et al., 2012). They emphasize that low quality or lacking cost evidence forces assumptions and excluding cost categories, resulting in incomplete and potentially misleading results. There is also lacking evidence for water supply programs (Hunter et al., 2009). The few studies with primary cost data for sanitation and hygiene behavior-change omit management and other software costs, use broad assumptions to fill data gaps, rely on recall by few respondents, and sample non-representative respondents, all problems that the authors acknowledge (Borghi et al., 2002, Briceño and Chase, 2015, Burr and Fonseca, 2011, Evans et al., 2009, Robinson, 2005, Trémolet et al., 2010). Importantly, these studies all use TDC methods. One study presents a BUC analysis of a WaSH program (Briceño and Chase, 2015). However, data were non-representative, costs were not disaggregated beyond program and household, and some local investments were omitted. Another study presents costs and benefits of latrine construction (Dickinson et al., 2015), though they focused on household costs.

We performed a BUC process and cost analysis of four CLTS interventions in Ghana and Ethiopia. We chose to present costs per population *targeted* rather than per population *reached* to focus this paper on our costing methods, findings, and implications. Converting to cost per population reached adds another layer of complexity, as there are multiple reasonable outcomes that can be used for this conversion, and the number of households reaching any given outcome depends on context. However, it is fine to convert to cost per household reached, which is done by dividing by the program costs by the percent of program households that reached the desired outcome.

We disaggregated results by intervention, geographic area, actor, time, and cost category to enable assessment of what drives variability, and how costs would transfer to other programs and settings. This study was implementation research conducted by Plan International USA and The Water Institute at UNC.

## 2 Methods

### 2.1 Program description

The four interventions were: in Ghana, (1) NGO-facilitated CLTS, and (2) NGO-facilitated CLTS with additional training for natural leaders; and in Ethiopia, (3) health extension worker (HEW) and kebele leader-facilitated CLTS, and (4) teacher-facilitated CLTS. Natural leaders are motivated community members who encourage others to construct and use latrines. A kebele is the lowest administrative unit in Ethiopia, comprising approximately 20–30 villages and 5000 people in rural areas. Implementation activities, actors, and timeline for the four interventions analyzed here are in the supplement. Project evaluations and implementation narratives are presented elsewhere (Crocker et al., 2017, Crocker et al., 2016a, Crocker et al., 2016b, Plan International Ethiopia, 2015, Plan International Ghana, 2015). Facilitation has three stages: *pre-triggering* - building a rapport and buy-in with community members; *triggering* - meeting with communities to conduct group activities that elicit emotional reactions, such as shame and disgust, to generate motivation to eliminate OD; and *follow-up* - monitoring a community's progress and guiding them toward eliminating OD. Further details on the CLTS approach can be found in the CLTS Handbook (Kar and Chambers, 2008).

For all four interventions, implementation began with an orientation workshop for district government officials. For intervention 1 (Ghana), implementation proceeded with CLTS facilitation by Plan International and local NGO (LNGO) staff (Table 1) with no formal training of local actors. Henceforth, Plan International and their contracted LNGOs are referred to as “Plan”. Intervention 2 (Ghana) included all the activities of intervention 1, with the addition of Plan training natural leaders to support CLTS. For interventions 3 and 4 (Ethiopia), Plan trained kebele leaders, and either HEWs or teachers, as facilitators. LNGOs were not contracted in Ethiopia. The four CLTS interventions cover a range of implementation arrangements and modalities as practiced in other organizations and countries (Venkataramanan, 2016, Venkataramanan, 2012), so the findings are relevant beyond this project.

**Table 1:** Plan implementation activities for four CLTS interventions in Ghana and Ethiopia.

Category	Activity	Ghana		Ethiopia	
		NGO CLTS	NGO CLTS + NL training	HEW CLTS	Teacher CLTS
Management	Project management	•	•	•	•
Training	District government orientation	•	•	•	•
	Training kebele leaders			•	•
	Training HEWs			•	
	Training teachers				•
	Training natural leaders		•	•	•
Facilitation	Facilitation	•	•	•	•
	Monitoring	•	•	•	•
	ODF celebration	•	•	•	•

Abbreviations: NGO, non-governmental organization; CLTS, community-led total sanitation; NL, natural leader; HEW, health extension worker; ODF, open-defecation free.

### 2.2 Context

Pre-existing factors in Ghana and Ethiopia enabled the interventions: national government had included CLTS in policy and established support mechanisms such as coordinating committees, and Plan had prior experience implementing CLTS and partnering with national and sub-national government. This study does not include costs associated with building government support for CLTS. Country economics also affect costs. Gross domestic product (GDP) per capita in 2015 was $1370 in Ghana and $619 in Ethiopia (The World Bank, 2017). Minimum wage at the project outset was $3.19 per day in Ghana, and $1.07 per day in Ethiopia.

### 2.3 Data collection and management

We developed new data collection tools to track implementation activities from the national- to village-level and to estimate local actor and community member activity, including checklists for management activities, training, and facilitation, and surveys for local actors and households (in the supplement). Checklists were developed by UNC researchers and Plan field staff, built on Plan's previous tools, and were designed to be simple and quick to fill out to maximize compliance and consistency.

Local actors and households were surveyed on their interactions, activities, and latrine spending. These surveys, which were also used to evaluate the outcomes of the interventions, are described in more detail in prior publications (Crocker et al., 2016a, Crocker et al., 2016b). Discussions with Plan staff three times per year clarified details and provided additional data. Unit costs including staff salaries, vehicle purchases, training venue rental, accommodation and meals, per-diems, and district government contracts were extracted from Plan's quarterly financial reports and discussions with staff. Web resources and literature were reviewed for general parameters such as official exchange rates and national minimum wages.

Checklist data were entered into Microsoft Access 2013, and checked for errors and gaps, which were corrected through correspondence with Plan staff. Surveys were analyzed in STATA SE13. Costs were calculated in Microsoft Excel 2013.

### 2.4 Analysis

Costs were categorized as program costs (management, training, facilitation) and local investments (local actor time, community member time, latrine spending). These categories were further split into components that could be calculated or estimated (Table 2). “Local investments” are those made by local actors, which includes sub-national government and natural leaders in both Ghana and Ethiopia; and kebele leaders, teachers, and HEWs in just Ethiopia.

**Table 2:** Cost categories and components.

Category		Components
Program costs	Management	Paid time – manager
		Paid time – field staff
		Office rent
		Office supplies
	Training^a^	Paid time – trainers
		Transportation
		Venue, accommodation, meals
		Per-diems
	Facilitation^b^	Paid time – Plan staff
		Paid time – government officials
		Transportation
		Per-diems
		ODF celebration costs
Local investments	Local actor time	Unpaid time – during training
		Unpaid time – traveling to training
		Unpaid time – during Plan's visits
		Unpaid time – in Plan's absence
	Community activity	Unpaid time – during Plan's visits
		Unpaid time – in Plan's absence
		Unpaid time – latrine construction
	Latrine spending^c^	Hired labor
		Purchased materials

a All activities outside villages that included local actors or community members. Includes orientations, training, and review meetings.

b Costs borne by Plan for activities within villages and kebeles.

c Excludes spending on communal latrines.

Paid time was calculated by multiplying hours spent on CLTS by hourly pay. Time in training and facilitation was aggregated from checklists, and allocated to intervention, region, actor, and month using metadata. Travel time was estimated using checklist data, Google Earth, and discussions with Plan staff. Management time was estimated from a checklist given to Plan staff after the interventions ended. When actors were not paid hourly, a 50-week/2000-hour work-year, 40-hour work-week, and 8-hour work-day were assumed.

Plan transportation costs were estimated using the American Automobile Association (AAA) guidelines (AAA, 2015), using intervention- and study site-specific parameters (details in supplement). Plan reimbursed trainees at a flat rate for transportation, which was used to calculate their transport costs.

Costs for office rent and supplies, training venue rental, and training materials were extracted from financial records, and allocated based on implementation activities. Unit costs for accommodation and meals for training attendees, and per-diems for Plan and government staff during village visits, were multiplied by person-days in training and in the field.

Spending on latrine construction (hired labor and purchased materials) was self-reported in household surveys. In Ghana, household-reported latrine age was used to identify latrines built during the CLTS interventions; in Ethiopia, baseline survey data was used. Since latrine age was not measured in Ethiopia, total latrine spending was distributed evenly across project months.

Unpaid time was monetized using estimated value-of-time for each actor. Unpaid time does not represent a financial investment; however, we included it as it is necessary for implementing CLTS, and some consider it a software cost (Whittington et al., 2012). Local actor and community member time engaged in CLTS activities and constructing latrines (including pit-digging) was calculated from checklists filled out by Plan when they were present, and otherwise estimated from survey responses. Local government, HEW, and teacher wages were used as their value-of-time, since they were fully employed. The national minimum wage and a value-of-time to minimum wage ratio of 0.5 were used for natural leaders and community members.

A sensitivity analysis for each estimated parameter was performed by calculating the change in cost associated with a ± 50% change in parameter values. The full analysis framework, unit costs and sources, and sensitivity analysis are in the supplement.

## 3 Results

Population numbers and implementation details are in Table 3. In Ghana, Plan's efforts focused on facilitating CLTS themselves, which involved frequent visits to project villages, whereas in Ethiopia Plan's efforts focused on training local actors as facilitators and thus Plan staff spent less time in villages. The program costs and local investments that follow are presented per household *targeted*.

**Table 3:** Descriptive statistics for villages receiving four CLTS interventions.

Variable	Ghana		Ethiopia	
	NGO CLTS	NGO CLTS + NL training	HEW CLTS	Teacher CLTS
Regions	3	3	2	2
Kebeles	–	–	2	4
Villages	29	29	54	111
Households	3443	3312	1624	3838
Population	14,269	12,936	9829	21,724
Average household size (people)	4.1	3.9	6.1	5.7
Total Plan village visits	350	375	11	22
Kebele leaders, HEWs, and teachers trained	–	–	20	76
Natural leaders trained	0	230	51	113
Households building latrines during CLTS	7%	15%	21%	17%

Abbreviations: NGO, non-governmental organization; CLTS, community-led total sanitation; NL, natural leader; HEW, health extension worker.

### 3.1 Program costs

**Fig. 1:**
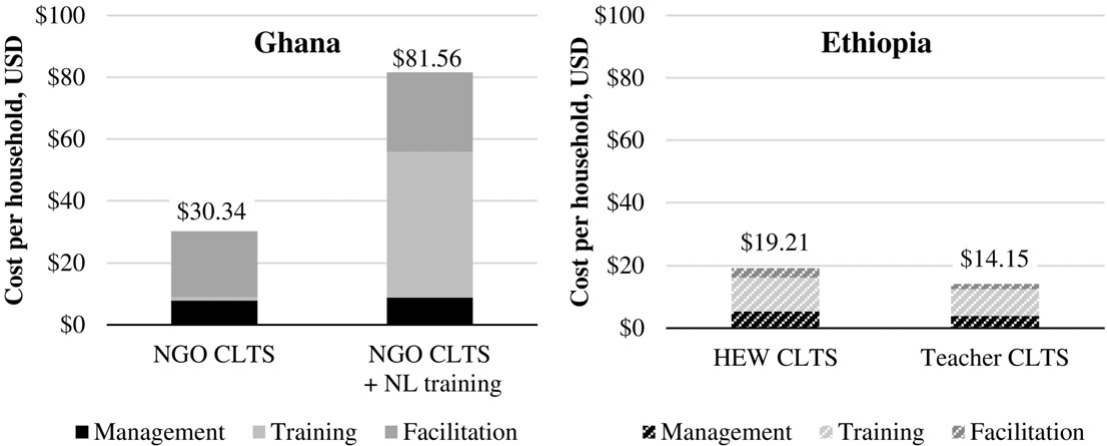
Program cost of four CLTS interventions in Ghana and Ethiopia, per household targeted. Abbreviations: NGO, non-governmental organization; CLTS, community-led total sanitation; NL, natural leader; HEW, health extension worker. All costs in this figure were borne by Plan. Costs are for the entire period of implementation.

Program costs (Fig. 1) are broken into management, training, and facilitation. In Ghana, NGO-facilitated CLTS cost $30.34 per household, rising to $81.56 when natural leader training was added. In Ethiopia, HEW-facilitated CLTS cost $19.21 per household targeted, dropping to $14.15 for teacher-facilitated CLTS.

Training was 4% of the program cost for NGO-facilitated CLTS in Ghana (training district government at 1-day orientation meetings), rising to 58% of program cost where natural leaders were trained (Table 4). Most of program cost in Ethiopia was training, where local actors were trained as facilitators. Further disaggregated costs (by region, intervention, and month), and normalized per-intervention and per-village, are in the supplement. The cost of training Plan staff is not included here, as in both countries they were already trained in CLTS at the project outset.

**Table 4:** Breakdown of program costs for four CLTS interventions in Ghana and Ethiopia.

Country	Intervention	Management	Training	Facilitation
Ghana	NGO CLTS	26%	4%	70%
	NGO CLTS + NL training	11%	58%	31%
Ethiopia	HEW CLTS	28%	56%	16%
	Teacher CLTS	27%	61%	11%

Abbreviations: NGO, non-governmental organization; CLTS, community-led total sanitation; NL, natural leader; HEW, health extension worker.

**Fig. 2:**
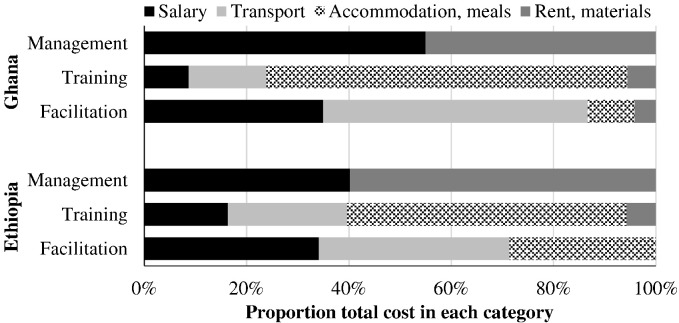
Components of program cost categories for community-led total sanitation interventions in Ghana and Ethiopia. All costs in this figure were borne by Plan.

Salary, transport, accommodation and meals, and rent and purchased materials contributions to cost categories are presented in Fig. 2. Management costs are split between salaries and office expenses. The cost of training local actors is dominated by accommodation and meals for the trainers and trainees, followed by transportation. Transportation is the principal facilitation cost, followed by salaries.

Sensitivity analysis of estimated parameters revealed that program costs are most sensitive to changes in travel time to project villages. Changing travel times by ± 50% results in up to a 12.4% change in program cost in Ghana, and 5.4% in Ethiopia.

### 3.2 Local investments

**Fig. 3:**
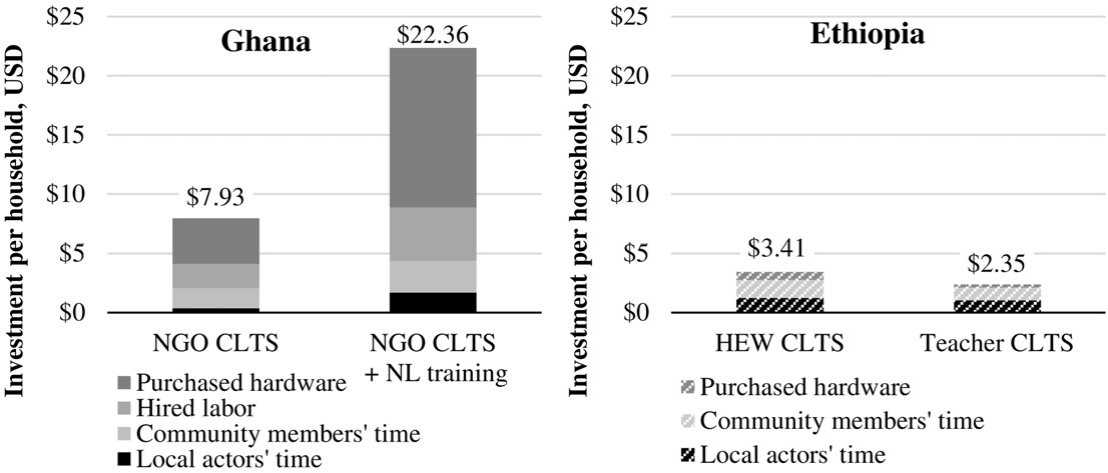
Local actor and community investments for four CLTS interventions in Ghana and Ethiopia, per household targeted. Abbreviations: NGO, non-governmental organization; CLTS, community-led total sanitation; NL, natural leader; HEW, health extension worker. All costs in this figure were borne by local actors and community members. Local actor and community member time was unpaid, and monetized using value-of-time assumptions. Hired labor and purchased hardware are household expenditures.

The financial costs (hired labor and purchased hardware) and unpaid value-of-time for local actors and community members are shown in Fig. 3. The aggregate local investment in Ghana was $7.93 per household targeted in villages receiving NGO-facilitated CLTS, and a substantially higher $22.36 in villages where natural leaders were trained, due both to more households building latrines (Table 3), and higher spending per latrine.

The aggregate local investment in Ethiopia was $3.41 per household targeted in villages receiving HEW-facilitated CLTS, and a lower $2.35 where teachers facilitated. The difference was due to teacher-facilitated CLTS being associated with lower attendance at community meetings, and fewer households constructing latrines (Table 3). Expenditures on latrines per household targeted were over 30 times higher in Ghana than Ethiopia. This dramatic difference was mostly due to households in Ghana purchasing materials for latrine construction (e.g. cement, wood, PVC) while nearly all households in Ethiopia built latrines from free materials (e.g. sticks, mud), and partly due to households in Ghana hiring labor to help with latrine construction, while no households in Ethiopia hired labor. Additional details on latrine construction and other outcomes are reported in prior publications.

In Ghana, hired labor for latrine construction was approximately one-quarter and purchased latrine materials approximately one-half of local investments (Table 5). In Ethiopia, where household spending on latrines was much lower, the value-of-time for local actors and community members exceeded 80% of local investments.

**Table 5:** Breakdown of local investments for four CLTS interventions in Ghana and Ethiopia.

Country	Intervention	Local actors' time	Community members' time	Hired labor	Purchased hardware
Ghana	NGO CLTS	5%	21%	26%	48%
	NGO CLTS + NL training	7%	12%	20%	60%
Ethiopia	HEW CLTS	35%	46%	0%	19%
	Teacher CLTS	43%	46%	0%	11%

Abbreviations: NGO, non-governmental organization; CLTS, community-led total sanitation; NL, natural leader; HEW, health extension worker.

Sensitivity analysis of estimated parameters revealed that local investments were most sensitive to changes in value-of-time for local actors. Changing value-of-time estimates by ± 50% results in a change in local investments of up to 9.7% in Ghana, and 42.9% in Ethiopia.

### 3.3 Time contributions to CLTS

In villages in Ghana that received only NGO-facilitated CLTS (the one intervention with no local actor training), for each hour that Plan spent on CLTS, local actors contributed 0.5 h, and community members contributed 5.9 h (Table 6). In villages in Ghana where Plan trained natural leaders, both local actor and community member time on CLTS increased (to 2.5 and 7.5 h for each hour of Plan's time).

**Table 6:** Time contributed to CLTS by different actors, and ratio of Plan's time to local actors' and community members' time, for four CLTS interventions in Ghana and Ethiopia.

Country	Approach	Full-time equivalent/10,000 people^a^			Ratio of total Plan hours to:	
		Plan	Local actors	Community	Local actor hours^b^	Community hours^c^
Ghana	NGO CLTS	1.4	0.7	8.4	1 to 0.5	1 to 5.9
	NGO CLTS + NL training	2.0	5.5	15	1 to 2.8	1 to 7.5
Ethiopia	HEW CLTS	0.7	3.3	19	1 to 4.7	1 to 27
	Teacher CLTS	0.5	3.4	14	1 to 6.8	1 to 28

Abbreviations: NGO, non-governmental organization; CLTS, community-led total sanitation; NL, natural leader; HEW, health extension worker.

a Full-time equivalent is 40 h per week.

b Local actors includes local government and NLs in both countries, and kebele leaders, HEWs, and teachers in Ethiopia.

c Community includes hired labor for latrine construction, and all other community activity related to CLTS.

In Ethiopia, for every hour of Plan's time, local actors contributed 4.7–6.8 h to CLTS—higher than in Ghana (Table 6). Community members contributed 27–28 h to CLTS per hour spent by Plan—over triple the ratio in Ghana. Plan staff in Ethiopia spent far more time training local actors than they did facilitating within villages, in contrast to Ghana where Plan's focus was on facilitation within villages. Plan's focus on training in Ethiopia resulted in Plan spending less time on CLTS per 10,000 people targeted than in Ghana.

Individually, trained local actors contributed 1.1–4.6 h per-week to CLTS on average (Table 7). Kebele leaders in Ethiopia were the most active, contributing up to 4.6 h per week or 12% full-time equivalent (FTE) to CLTS. On average, community members spent far less time than local actors on CLTS (under 6 min per week individually). However, this belies the fact that not every community member spends time on CLTS. Community members also form the biggest group of actors, who collectively spent the most time on CLTS (Table 6).

**Table 7:** Time spent on CLTS implementation by local actors and community members.

Country	Approach	Average hours per-person per-week^a^ (FTE^b^)					
		Govt.	KLs	HEWs	Teachers	NLs	Community
Ghana	NGO CLTS	2.5^a^ (6.2%)^b^	–	–	–	0.18 (0.45%)	0.04 (0.09%)
	NGO CLTS + NL training		–	–	–	1.5 (3.7%)	0.07 (0.16%)
Ethiopia	HEW CLTS	1.1 (2.6%)	2.9 (7.2%)	3.4 (8.5%)	–	–	0.09 (0.23%)
	Teacher CLTS		4.6 (12%)	–	2.2 (5.4%)	–	0.07 (0.17%)

Abbreviations: NGO, non-governmental organization; CLTS, community-led total sanitation; KL, kebele leader; NL, natural leader; HEW, health extension worker; FTE, full-time equivalent.

a Average hours-per-week spent on CLTS per person.

b Full-time equivalent is 40 h per week.

**Fig. 4:**
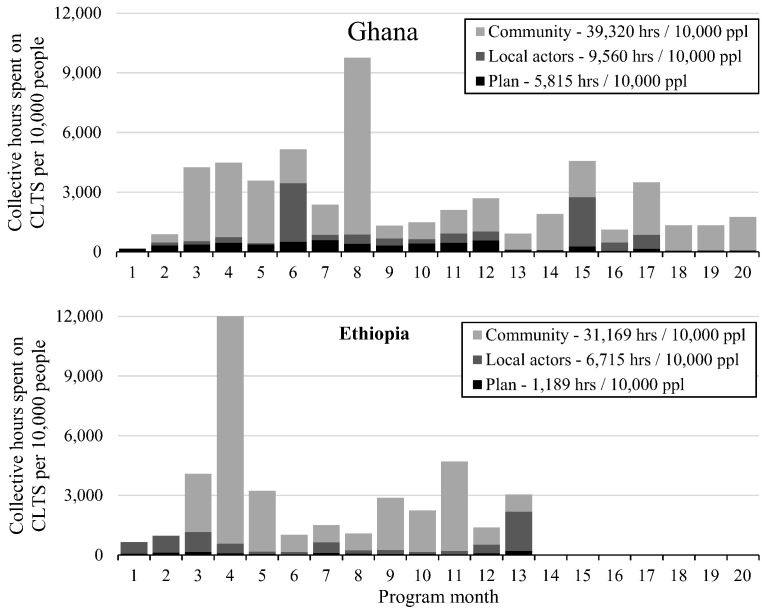
Hours spent on CLTS by different actors per month, Ghana and Ethiopia. Abbreviations: CLTS, community-led total sanitation. Plan activities ended in month 20 in Ghana, and in month 13 in Ethiopia. Local actor and community member activity likely continued after Plan activities ended, but was not tracked.

In Ghana, Plan's time spent on CLTS declined after 12 months (Fig. 4), when LNGO contracts ended and the number of facilitators dropped from 16 to 4. Local actor time peaked during training in months 6 and 15. Community activity peaked during triggering in months 3–5. The peak at month 8 is because many households reported constructing their latrine “one year ago” on a survey in month 20, thus activity shown in month 8 likely occurred across multiple months.

In Ethiopia, Plan's time spent on CLTS was much lower than in Ghana, and was more evenly distributed. Plan and local actor activity peaked during training in months 2, 3, and 13. Community activity peaked during triggering in month 4, and during ODF celebrations in months 9–11.

## 4 Discussion

### 4.1 Program costs

We present costs per household targeted in this paper. Cost per household *reached* would be higher, as WaSH programs typically do not reach all targeted beneficiaries.^1^ In Ghana, the cost of implementing NGO-facilitated CLTS was $30.34 per household targeted, 70% of which was from facilitation. The addition of natural leader training raised costs to $81.56 per household targeted. This substantial difference was mostly due to expensive accommodation and meals at training venues, which together were 70% of training costs. There were no low-cost hotels capable of holding 80 natural leaders available.

In Ethiopia, the cost of implementing HEW-facilitated CLTS was $19.21 per household targeted, which dropped to $14.15 for teacher-facilitated CLTS. Management and training costs were lower for the teacher-facilitated approach. Only one kebele in each region received HEW-facilitated CLTS. However, teachers were grouped together from two kebeles in each region for training, which, on a per kebele basis, lowered management costs for planning training, venue rental cost, and trainer costs. Facilitation cost was lower on average in teacher-facilitated CLTS kebeles because two of four teacher-facilitated kebeles were not verified as ODF, eliminating the cost of ODF celebrations (not a desirable cost reduction).

Program costs in Ghana were over four times those in Ethiopia on average. Management and training costs in Ghana were approximately double those in Ethiopia. Facilitation costs in Ghana were over ten times those in Ethiopia, because Plan trained local actors as facilitators in Ethiopia rather than leading facilitation themselves. In contrast, in Ghana, NGO staff led facilitation within villages. The program cost differences demonstrate how implementation arrangements can determine costs, and thus how many people can be reached within a given budget. Given that effectiveness also varies between interventions, we would expect cost-effectiveness to vary even more than costs.

Despite dramatic differences in absolute costs between countries, relative costs were similar in meaningful ways. Management was 26–28% of program cost for three of four interventions (excepting CLTS with natural leader training in Ghana, in which accommodation and meals for training drove up total costs, reducing the management proportion). For the three interventions that included local actor training, training cost was 56–61% of program cost. In both Ghana and Ethiopia, over half of training cost came from accommodation and meals, with trainee transport as the next largest portion. The relative cost of management and training may reflect relative costs of other software-heavy behavior-change approaches.

The largest contributor to facilitation cost was transportation in both Ghana and Ethiopia. CLTS projects often occur in remote areas and difficult to access villages. This was the case for the interventions analyzed here, which greatly impacted project costs, due to rough roads that necessitate expensive four-wheel drive vehicles, high fuel prices, and facilitators spending significant time traveling to and from project villages. However, these same villages may be most appropriate for CLTS, as other WaSH projects often do not reach them, and CLTS is most effective in these settings (Crocker et al., 2016a).

Training costs are subject to economies of scale, as venue rental and trainer pay would not increase with moderate increases in trainees. Total costs would also be lower where training venues are cheaper or closer to project villages. Management costs are also subject to economies of scale, provided that interventions are implemented with some degree of consistency across all project villages. Facilitation costs would be minimally if at all subject to economies of scale, as facilitation activities are proportional to the population targeted.

### 4.2 Local investments

During NGO-facilitated CLTS in Ghana, local actors and community members invested time and money worth $7.93 per household targeted, which rose to $22.36 where natural leaders were trained. Household spending contributed approximately 75% in both cases, demonstrating that training natural leaders increased household investment in latrines. Local investments were much lower in Ethiopia: $3.41 per household targeted for HEW-facilitated CLTS, and $2.35 for teacher-facilitated CLTS. Most of the difference in local investments between countries was from very low spending on latrines in Ethiopia: $0.38 per household targeted, compared to $11.81 in Ghana, and some of the difference was due to lower value-of-time in Ethiopia where wages are lower. Latrines in Ethiopia were built mostly of free, low-durability local materials. Local investments (by local actors and community members) could also be call program outputs, as a beneficial and voluntary response to CLTS.

The three interventions in which Plan trained local actors demonstrated that training leveraged local actors to facilitate or support CLTS. Where Plan trained local actors, for each hour that Plan spent on CLTS, local actors contributed 2.8 h in Ghana, and 4.7–6.8 h in Ethiopia. For the one intervention that did not include training local actors (NGO-facilitated CLTS in Ghana), local actors spent half as much time as Plan on CLTS.

Collectively, community members contributed the most time to CLTS, unsurprisingly given they are the targeted beneficiaries, and that CLTS is a participatory approach. Each hour Plan spent on CLTS led to community members contributing 5.9–7.5 h in Ghana, and 27–28 h in Ethiopia. The higher ratios in Ethiopia do not represent a significantly higher level of activity by local actors or communities; rather they represent Plan generating the same level of local activity with less of their own time. This efficiency is due to the interventions in Ethiopia focusing on training local actors as facilitators.

Individually, trained local actors spent 2.6%–12% FTE supporting CLTS facilitation, with kebele leaders and HEWs in Ethiopia committing the most time. While CLTS leverages investment of time by local actors, it also burdens them. HEWs have many other job responsibilities. Kebele leaders are not compensated for time spent on CLTS. CLTS also leverages investment of time and money by community members. The time-burden on community members was much lower than on local actors, and spending on latrines was voluntary.

### 4.3 Research in context

Four TDC studies have reported costs of sanitation promotion programs. The first TDC study reported that WaterAid CLTS programs in Bangladesh, Nepal, and Nigeria cost $6–84 per household targeted, but overhead costs were underreported and underestimated, the three countries had non-compatible financial tracking, and disaggregated costs were “indicative” due to their TDC method (Evans et al., 2009). The second TDC study did not describe data collection or cost analysis methods, and reported government-facilitated CLTS costing $1 per household *reached* in Ethiopia (costs per household targeted would be even lower) (Sah and Negussie, 2009). The third TDC study reported non-CLTS sanitation promotion as costing $2–45 per household targeted in three countries in South Asia, although methodological deficiencies are present, such as excluding paid government time despite government facilitating the project (Robinson, 2005). The fourth TDC study reported software of non-CLTS sanitation promotion as costing $7–144 per household reached in six countries. Costs per household targeted were not reported, data collection was not described, and software cost components were unclear (Trémolet et al., 2010).

We found one BUC sanitation study, which reported that World Bank-funded government-facilitated CLTS in Tanzania cost $30 per household targeted, and $50 when hygiene promotion was included (Briceño and Chase, 2015). Because this was a BUC study, it may be interpreted as more accurate and comprehensive than the TDC studies. However, it used recall-based data, a non-representative sample of respondents, and program costs were not disaggregated.

These prior studies exclude some cost categories, neglect local investments, or use inappropriate TDC methods and non-representative respondents, yielding inaccurate and underestimated costs. This makes comparison to our results difficult, as we used BUC and cover all program costs and local investments. The low-end of the cost-range for all the TDC studies ($6, $1, $2, and $7) was dramatically lower than for the one prior BUC study ($30), and for our study ($14). While this represents a small number of studies and covers different interventions implemented in different countries, it does support our hypothesis that TDC methods will underestimate the cost of WASH programs, particularly those that are participatory or include behavior-change activities. The cost per household targeted range in our study overlaps with the range of four of the five studies mentioned above.

When comparing our results to those of other studies, it is important to note that program costs and local investments are influenced by social and political factors (in addition to being influenced by implementation arrangements, as discussed previously). In Ethiopia, every kebele is supposed to have a staffed health post and school, which enabled Plan approach to train facilitators, resulting in lower costs than in Ghana. Ethiopia has a weaker economy, lower GDP, and lower wages than in Ghana, which resulted in lower costs for Plan, and value-of-time for local actors and community members, which made both program costs and local investments lower than in Ghana. This may also explain the lower expenditures on latrines in Ethiopia.

### 4.4 Contributions

This is the first study to provide comprehensive, accurately tracked, disaggregated costs for any WaSH behavior-change program. This study provides insight into the cost of project management, training, and facilitation, all of which are ubiquitous among participatory behavior-change public health projects, making these findings relevant beyond WaSH. The evidence provided by this study is particularly important given that the SDGs emphasize capacity building, local participation, and public finance to leverage other investments such as local actor and community investments (UN General Assembly, 2015), all of which are present in the interventions analyzed here.

We developed data collection tools and a framework for cost analysis of complex programs that are implemented by multiple organizations, are flexible and adaptable, involve cross subsidies, or include local investments. As these program characteristics are common beyond WaSH, these tools are relevant for other public health programs. For example, interventions to influence use of clean cookstoves, postnatal health behaviors, and HIV prevention behaviors are all common public health behavior-change interventions that contain the above characteristics.

### 4.5 Limitations

Our findings are context specific, as costs vary between settings and interventions. However, this study covered four interventions across five regions in two countries, and presents disaggregated results, to show how costs vary by setting and intervention, and how implementation activities drive cost variation. Some local investments were estimated from survey data. Potential survey sampling error and bias were minimized by a large, representative sample, and experienced contractors. Local investments were sensitive to value-of-time estimates, and may be underestimated given the 0.5 value-of-time to minimum wage ratio we used. Transport cost calculations rely on assumptions for vehicle depreciation, maintenance, travel time, and driving speed. Assumptions were based on real data for vehicles used, and AAA travel cost models, which may underestimate maintenance and fuel costs for travel on rough roads. A sensitivity analysis was conducted on value-of-time and transportation parameters to test their robustness. For contracted work (LNGO facilitation, and district government monitoring), cost allocation to management, salary, and transport categories was based on submitted budgets, which may deviate from exact expenditures by category. However, the total contract costs are accurate, as they reflect payments. Local investments likely continued after cost tracking as part of this study stopped (in contrast to program costs, which did not continue).

## 5 Conclusions

This is the first study to present comprehensive, accurate, disaggregated costs of a WaSH behavior-change program. These findings can add value to WaSH policy and planning discussions, and can be incorporated into cost-effectiveness research as the first reliable cost figures for a CLTS intervention. Evidence on process and costs is an important resource for policy and funding decisions, program design and management, cost-effectiveness and cost-benefit studies, modeling program scale up, and evaluating and comparing different interventions.

This study offers a robust method and process for tracking and calculating costs, which can be followed and further developed to build evidence on a broad range of WaSH interventions that is consistent and accurate. The multi-site, multi-intervention research approach used in this study is important for understanding how findings would transfer to other programs and settings.

## Competing interests

DS is employed by Plan International USA, and oversaw the interventions evaluated. The other authors declare no real or potential conflicts of interest.

## Appendix A Supplementary data

Supplementary tables and figures. Image 1

Supplementary data and analysis - Ghana. Image 2

Supplementary data and analysis - Ethiopia. Image 3

## Footnotes

1 Cost per population reached could be five times higher or more in this case, given the low percent of the target population that changed from open defecation to latrine use (Crocker et al., 2017). However, converting to average cost per household reached can be misleading, as various outcomes can be chosen, and outcomes vary between settings.
