# Supplementary material for: The true costs of participatory sanitation: Evidence from community-led total sanitation studies in Ghana and Ethiopia
Source: Sci Total Environ. 2017 Dec 1;601-602:1075–83. doi: 10.1016/j.scitotenv.2017.05.279 (PMC5536257; doi:10.1016/j.scitotenv.2017.05.279)
Supplement: Supplementary file 1 — Supplementary tables and figures. [file mmc1.docx]

**SUPPLEMENTAL MATERIAL**

**The true costs of participatory sanitation: evidence from community-led total sanitation studies in Ghana and Ethiopia**

Jonny Crocker, Darren Saywell, Katherine F Shields, Pete Kolsky, Jamie Bartram

**Table of contents**

**Table S1.** Implementation timeline for four CLTS interventions in Ghana and Ethiopia

**Table S2.** Project activities and responsible parties for four CLTS interventions in Ghana and Ethiopia

**Table S3.** Cost categories, data sources, and data descriptions

**Table S4.** Unit costs and data sources

**Table S5.** Disaggregated program costs and local investments

**Table S6.** Analysis of cost sensitivity to estimated parameters

**Table S7.** Management data collection checklist

**Table S8.** Training data collection checklist

**Table S9.** Facilitation data collection checklist

**Table S1.** Implementation timeline for two CLTS interventions in Ghana

| **Period** |  | **NGO facilitated CLTS + NL training** | **NGO facilitated CLTS** |
| --- | --- | --- | --- |
| Nov 2012 - Jan 2013 |  | District orientation | District orientation |
| Oct 2012 - Jan 2013 |  | Pre-triggering | Pre-triggering |
| Dec 2012 - Mar 2013 |  | Triggering | Triggering |
| Mar 2013 |  | - | Natural leader training |
| Jan 2013 - Mar 2014 |  | Follow-up | Follow-up |
| May 2013 |  | - | Natural leader review meeting |
| Sep 2013 |  | - | Natural leader review meeting |
| Sep - Dec 2013 |  | ODF certification and celebrations | ODF certification and celebrations |
| Dec 2013 |  | - | Natural leader refresher training |
| Feb 2014 |  | - | Natural leader review meeting |

**Table S2.** Implementation timeline for two CLTS interventions in Ethiopia

| **Period** |  | **HEW facilitated CLTS** | **Teacher facilitated CLTS** |
| --- | --- | --- | --- |
| Sep 2012 |  | District orientation | District orientation |
| Oct 2012 |  | Pre-triggering | Pre-triggering |
| Nov 2012 |  | HEW and kebele leader training | Teacher and kebele leader training |
| Nov 2012 - Jan 2013 |  | Triggering | Triggering |
| Dec 2012 - May 2013 |  | Follow-up | Follow-up |
| Mar 2013 |  | HEW and kebele leader review meeting | Teacher review meeting |
| Jun - Nov 2013 |  | ODF certification and celebrations | ODF certification and celebrations |
| Sep 2013 |  | Natural leader training | Natural leader training |

**Table S3.** Project activities and responsible parties for four CLTS interventions in Ghana and Ethiopia.

|  | **Ghana** | | **Ethiopia** | |
| --- | --- | --- | --- | --- |
| **Activity** | **NGO CLTS** | **NGO CLTS  + NL training** | **HEW CLTS** | **Teacher CLTS** |
| Project management | Plan | Plan | Plan | Plan |
|  | LNGO | LNGO |  |  |
| District government orientation | Plan | Plan | Plan | Plan |
| Training kebele leaders | - | - | Plan | Plan |
| Training HEWs | - | - | Plan | - |
| Training teachers | - | - | - | Plan |
| Training natural leaders | - | Plan | Plan | Plan |
|  |  | Regional government |  |  |
| Attending trainings | - | Natural leaders | Kebele leaders | Teachers |
|  |  |  | HEWs | Kebele leaders |
|  |  |  | Natural leaders | Natural leaders |
| Pre-triggering community visits | Plan | Plan | Plan | Plan |
|  |  |  | HEWs | Teachers |
| Community triggering | LNGO | LNGO | HEWs | Teachers |
|  | Plan | Plan | Kebele leaders | Kebele leaders |
| Post-triggering follow-up visits | LNGO | LNGO | HEWs | Teachers |
|  | Plan | Plan | Kebele leaders | Kebele leaders |
|  | District government |  |  |  |
| Sanitation status monitoring | Natural leaders | Natural leaders | HEWs | Teachers |
|  | LNGO | LNGO | Kebele leaders | Students |
|  | District government | District government | District government | Kebele leaders |
|  | Plan | Plan | Plan | District government |
|  |  |  |  | Plan |
| ODF verification | District government | District government | Kebele leaders | Kebele leaders |
|  |  |  | HEWs | Teachers |
| ODF certification | Regional government | Regional government | District government | District government |
| Attending meetings | Community members | Community members | Community members | Community members |
|  | Natural leaders | Natural leaders | Kebele leaders | Teachers |
|  |  |  | HEWs | Kebele leaders |
|  |  |  | Natural leaders | Natural leaders |
| Home visits | Natural leaders | Natural leaders | Kebele leaders | Teachers |
|  | LNGO | LNGO | HEWs | Kebele leaders |
|  | Plan | Plan | Natural leaders | Natural leaders |
| Latrine construction | Community members | Community members | Community members | Community members |
|  | Natural leaders | Natural leaders |  |  |

**Table S4.** Cost categories, components, data sources, and data descriptions.

| **Cost category and component** | | **Data source** | **Data description** |
| --- | --- | --- | --- |
| **Management** | Paid time - manager | Management checklist | Time spent on different management activities |
|  |  | Financial data | Salary of project manager |
|  | Paid time - field staff | Management checklist | Time spent on different management activities |
|  |  | Financial data | Salary of field staff |
|  | Office rent | LNGO contracts | Funds allocated to office rent and utilities |
|  | Office supplies | Financial data | Cost of purchased office supplies |
| **Training** | Paid time - trainers (including travel time) | Checklists | Location of each training |
|  |  |  | Trainers present on each day |
|  |  |  | Days and hours / day in training |
|  |  | Discussions with project team, google earth | Travel distance and time to training venues |
|  |  | Financial data | NGO staff salaries (Plan) |
|  |  | Government contracts | Rate paid to government trainers |
|  | Transportation | Checklists | Number of transportation days |
|  |  | Discussions with project team, google earth | Travel distance and time to transport trainers and trainees |
|  |  | Financial data | Fixed reimbursement costs for trainee transportation |
|  |  |  | Purchased vehicle cost |
|  |  | Web search | Historical gas prices |
|  |  | American Auto Association (AAA) | Guideline and general parameters for transportation costing |
|  | Venue, accommodation, meals | Financial data | Amount paid |
|  |  | Discussions with project team | Daily rate paid for accommodation (Ethiopia only) |
|  | Per-diems | Checklists | Total person-days spent in training |
|  |  | Financial data | Total paid for per-diems per training event |
|  |  | Discussions with project team | Per-diem rate per person-day |

**Table S4** (continued). Cost categories, components, data sources, and data descriptions.

| **Category and sub-category** | | **Data source** | **Description** |
| --- | --- | --- | --- |
| **Facilitation** | Paid time - Plan and LNGO facilitators (including travel time) | Checklists | NGO staff present for each community visit |
|  |  |  | Duration of each community visit |
|  |  |  | Field days, and communities visited each day |
|  |  | Discussions with project team, google earth | Travel distance and time to project districts, and between communities |
|  |  | Financial data | Plan staff salaries |
|  |  | LNGO contracts | NGO staff salaries |
|  | Paid time - government officials | Checklists | Communities visited by government by date for contracted work |
|  |  | Government contracts | Contract amount for monitoring and ODF certification activities |
|  | Transportation | Checklists | Field days, and communities visited each day |
|  |  | Discussions with project team, google earth | Travel distance and time to project districts, and between communities |
|  |  | Financial data | Purchased vehicle cost |
|  |  | Web search | Historical gas prices |
|  |  | American Auto Association (AAA) | Guideline and general parameters for transportation costing |
|  | Per-diems | Checklists | Number of person-days in the field |
|  |  | Discussions with project time | Per-diem rate for field days |
|  | ODF celebration costs | Checklists | Communities that had ODF celebrations, and dates of celebrations |
|  |  | Financial data | Materials purchased and amount paid |
| **Local actor time** | *Unit costs - value of local actors' time* | Web search | Government employee wages and national minimum wages |
|  |  | Literature | Value-of-time to wage ratios |
|  | Unpaid time - during training | Checklists | Number of each local actor |
|  |  |  | Dates and duration of training sessions |
|  | Unpaid time - traveling to training and villages | Checklists | Location of each training |
|  |  |  | Local actors present on each day |
|  |  |  | Government present for community visits |
|  |  | Discussions with project team, google earth | Travel distance and time to training venues |
|  |  |  | Travel distance and time to project districts, and between communities |
|  | Unpaid time - during Plan and LNGO community visits | Checklists | Communities visited |
|  |  |  | Number of each local actor present |
|  |  |  | Duration of each community visit |
|  | Unpaid time - community meetings and home-visits in NGO's absence | Local actor surveys | Hours per month on CLTS activities |
|  |  | Checklists | Number of each local actor trained from each community |
|  |  | Household census and surveys | Population of each community |

**Table S4** (continued). Cost categories, components, data sources, and data descriptions.

| **Category and sub-category** | | **Data source** | **Description** |
| --- | --- | --- | --- |
| **Community activity** | *Unit cost - value of community members' time* | Web search | National minimum wages |
|  |  | Literature | Value-of-time to wage ratios |
|  | Unpaid time - during Plan and LNGO community visits | Checklists | Communities visited |
|  |  |  | Number of community members present during community visits |
|  |  |  | Duration of community visits |
|  |  | Household surveys | Percent of community attending CLTS meetings |
|  |  | Discussions and review meetings with project team | Filling in gaps regarding community attendance |
|  | Unpaid time - during local actor meetings and home-visits | Local actor surveys | Hours per month of local actor engagement with community members |
|  |  | Checklists | Number of each local actor trained from each community |
|  |  | Household surveys | Percent of community attending CLTS meetings |
|  | Unpaid time - latrine construction | Household surveys | Hours spent on constructing their latrine |
|  |  |  | Hours of unpaid help received for latrine construction |
| **Latrine spending** | Hired labor | Household surveys | Amount spent on hired labor for latrine construction |
|  | Purchased materials | Household surveys | Total amount spent on materials for latrine construction |
|  |  |  | Portion of latrines that were built during the CLTS interventions |

**Table S5**. Unit costs and data sources.

| **Parameter** | | **Value^*^** | **Notes** | **Source and assumptions** |
| --- | --- | --- | --- | --- |
| **Ghana** | |  |  |  |
| *Exact financial costs* | |  |  |  |
|  | Plan project manager | $8.34 / hour | Uses a 50-week, 2000-hour workyear assumption. | Plan financial records. |
|  | Plan project coordinator | $3.56 / hour | Uses a 50-week, 2000-hour workyear assumption. | Plan financial records. |
|  | Plan per-diem | $10.99 - $16.51 / day | For Plan staff on field days. Per-diem was paid at a fixed rate, variation is due to the changing exchange rate. |  |
|  | Plan office and supplies costs | $506.96 / month | For 58 villages, over an 18-month period | Plan financial records. |
|  | Training venue rental | $31.70 - $337.20 / day | Venue rental costs varied by region and town size. Smaller training sessions were held in district towns, which had lower rates than region capitals. | Plan financial records |
|  | Meals | $6.03 - $30.70 / person-day | During training. Meal costs varied by region and town size. | Plan financial records |
|  | Accommodation | $17.73 - $47.81 / night | Accommodation costs varied by region and town size. | Plan financial records |
|  | Trainee transport | $2.36 - $21.10 / trip | Trainees were reimbursed for transportation at fixed rates, depending on the distance and region | Plan financial records |
|  | Government contract for monitoring | $1,880 - $3,874 | For follow-up monitoring visits to 20 villages. Contract amounts vary by region. | Government contract budgets, Plan financial records |
|  | Government contract for ODF certification | $2,228 - $7,760 | For follow-up monitoring visits to 20 villages. Contract amounts vary by region. | Government contract budgets, Plan financial records |

**Table S5** (continued). Unit costs and data sources.

| **Parameter** | | **Value^*^** | **Notes** | **Source and assumptions** |
| --- | --- | --- | --- | --- |
| **Ghana** | |  |  |  |
| *Unit costs involving estimation or extrapolation* | | |  |  |
|  | Plan transport | $0.80 / mile | Toyota Hilux | Purchase cost ($37,410) - Plan Ghana financial records. Depreciation (15% first year), maintenance ($0.0565/mile), and tire cost ($0.0138/mile) assumptions from AAA "Your Driving costs" 2015 Edition. Miles per gallon (21.3) taken from Fuelly.com for 2012 Toyota Hilux. Historical gas prices in Ghana ($4/gallon) from the Trading Economics webpage. |
|  | Plan transport | $24.01 / hour | Toyota Hilux | Above value with an average 30 miles per hour assumption. |
|  | Plan transport | $0.33 / mile | Motorcycle | Purchase cost ($5,139) - Plan Ghana financial records. Depreciation (20% first year), maintenance ($0.0565/mile), and tire cost ($0.0138/mile) assumptions from AAA "Your Driving costs" 2015 Edition. Forty miles per gallon assumed. Gas prices ($4/gallon) from the Trading Economics webpage. |
|  | Plan transport | $9.94 / hour | Motorcycle | Above value with an average 30 miles per hour assumption. |
|  | Project management by local NGO | $310.08 - $413.44 / month | For 20 villages, over a 12-month contracts. Contract amounts vary by region. | Local NGO proposal budget, Plan financial records |
|  | Local NGO office rent, utilities, supplies | $363.44 - $457.36 / month | For 20 villages, over a 12-month contracts. Contract amounts vary by region. | Local NGO proposal budget, Plan financial records |
|  | Local NGO facilitator | $6.01 - $9.75 / hour | For 20 villages, over a 12-month contracts. Contract amounts vary by region. | Local NGO proposal budget, Plan financial records |
|  | Local NGO other field costs | $206.72 - $438.42 | For 20 villages, over a 12-month contracts. Contract amounts vary by region. | Local NGO proposal budget, Plan financial records |
|  | Transportation - Local NGO team | $64.65 - $102.93 / hour |  | Based on travel time and budget lines for a driver and transportation in local NGO proposals |
|  | District government official | $3.13 / hour | Average compensation rate used by Plan during training | Plan financial records |
|  |  |  |  |  |
| *Cost parameters with sources external to this project* | | |  |  |
|  | National minimum wage | $2.12 - $3.19 / day | Pre-2015 official minimum wage in Ghana. Wage varies with changing exchange rate. | US Department of State |
|  | Value-of-time to minimum wage ratio | 0.5 | Used for natural leaders and community members. | High end of range in Whittington (2012) and Jeuland (2010) |
|  | Laborer wage | $0.50 - $1 / hour | Low end used for Upper West only | Based on primary author's prior work in Ghana |
|  | GHS / USD exchange rate | 1.88 - 2.83 | The exchange from the first day of each month was used | XE.com |

**Table S5** (continued). Unit costs and data sources.

| **Parameter** | | **Value^*^** | **Notes** | **Source and assumptions** |
| --- | --- | --- | --- | --- |
| **Ethiopia** | |  |  |  |
| *Exact financial costs* | |  |  |  |
|  | Plan project manager | $5.64 / hour | Uses a 50-week, 2000-hour workyear assumption. | Plan financial records. |
|  | Plan project coordinator | $4.93 / hour | Uses a 50-week, 2000-hour workyear assumption. | Plan financial records. |
|  | Plan office and supplies costs | $1,083.54 / month | For 6 kebeles, over a 13-month period | Plan financial records. |
|  | Training venue rental | $29.01 - $105.67 / day | Costs varied by region and location. Venues were more expensive in the SNNP region. | Plan financial records |
|  | Meals | $1.32 - $10.61 / person-day | The low end was for training in kebeles. Meals were at the high end of the range for most training sessions. | Plan financial records |
|  | Accommodation | $7.13 - $7.54 / night | Trainees were reimbursed at a fixed rate. The range is due to changing exchange rate. | Plan financial records |
|  | Trainee transport | $7.13 - $7.54 / night | Trainees were reimbursed at a fixed rate. The range is due to changing exchange rate. | Plan financial records |
|  | ODF certification | $1,325.62 - $1690.53 / kebele | Includes per-diems for government verification team, and materials for ODF celebration | Plan financial records |
|  |  |  |  |  |
| *Unit costs involving estimation or extrapolation* | | |  |  |
|  | Plan transport | $1.18 / mile | Toyota Landcruiser | Purchase cost - Plan Ethiopia financial records. Depreciation (15% first year), maintenance ($0.0565/mile), and tire cost ($0.0138/mile) assumptions from AAA "Your Driving costs" 2015 Edition. Miles per gallon (13.8) taken from Fuelly.com for a 2012 Toyota Landcruiser. Historical gas prices in Ethiopia ($4/gallon) from the Trading Economics webpage. |
|  | Plan transport | $35.42 / hour | Toyota Landcruiser | Above value with an average 30 miles per hour assumption. |

**Table S5** (continued). Unit costs and data sources.

| **Parameter** | | **Value^*^** | **Notes** | **Source and assumptions** |
| --- | --- | --- | --- | --- |
| **Ethiopia** | |  |  |  |
| *Cost parameters with sources external to this project* | | |  |  |
|  | Health Extension Worker wage | $47.97- $50.71 / month | Used for health extension workers and teachers (who, by definition, were fully employed) | Interviews with district health officers during the situational assessment in 2012. (Crocker 2015) |
|  | Health extension worker supervisor | $65.14 - $68.87 / month | Used for district government and kebele leaders (who, by definition, were fully employed) | Interviews with district health officers during the situational assessment in 2012. |
|  | Public sector minimum wage | $22.19 - $23.46 / month | Used for natural leaders and community members. | US Department of State |
|  | Value-of-time to minimimum wage ratio | 0.5 | Used for natural leaders and community members. | High end of range in Whittington (2012) and Jeuland (2010) |
|  | ETB / USD exchange rate | 17.9 - 18.9 | The exchange from the first day of each month was used | XE.com |
| *Values are all presented in USD. Those that were originally in GHS were converted using the exchange rate for the first day of the month in which they occurred. | | | | |
| **References:** | |  |  |  |
| Whittington D, Jeuland MA, Barker K, Yuen Y. Setting priorities, targeting subsides among water, sanitation, and preventative health interventions in developing countries. World Dev. 2012;40(8):1546-1568. doi:10.1016/j.worlddev.2012.03.004. | | | | |
| Jeuland MA, Lucas M, Clemens J, Whittington D. Estimating the private benefits of vaccination against cholera in Beira, Mozambique: A travel cost approach. J Dev Econ. 2010;91(2):310-322. doi:10.1016/j.jdeveco.2009.06.007. | | | | |
| Trading Economics. Ghana gasoline prices. http://www.tradingeconomics.com/ghana/gasoline-prices. | | | |  |
| Bureau of Democracy, Human Rights and Labor, US Department of State. Country reports on human rights practices for 2014. http://www.state.gov/j/drl/rls/hrrpt/humanrightsreport/index.htm#wrapper | | | | |
| XE currency charts (USD/GHS). http://www.xe.com/currencycharts/?from=USD&to=GHS. | | | |  |
| Rajkumar AS, Gaukler C, Tilahun J. Combating malnutrition in Ethiopia: an evidence-based approach for sustained results. World Bank Publications. 2011. | | | | |
| Crocker J, Rowe R. Community-Led Total Sanitation in Ethiopia: Findings from a Situational Assessment. Chapel Hill, NC; 2015. http://waterinstitute.unc.edu/files/2015/03/situational-assessment-ethiopia-2015-02.pdf. | | | | |

**Table S6.** Disaggregated program costs and local investments.

| **Country** | **Region** | **Treatment** | **Program costs** | | | **Local investments** | | | **TOTAL** | **Notes** |
| --- | --- | --- | --- | --- | --- | --- | --- | --- | --- | --- |
|  |  |  | **Management** | **Training** | **Facilitation** | **Local actors** | **Community activity** | **Hardware** |  |  |
| Ghana | Central | NGO CLTS | $8,797 | $1,199 | $28,573 | $314 | $1,500 | $4,319 | $45,797 | 9 villages |
| Ghana | Central | NGO CLTS + NL training | $9,525 | $38,427 | $32,281 | $1,523 | $3,655 | $23,501 | $110,747 | 9 villages |
| Ghana | Upper West | NGO CLTS | $9,308 | $928 | $18,270 | $372 | $1,450 | $1,282 | $33,801 | 10 villages |
| Ghana | Upper West | NGO CLTS + NL training | $10,037 | $62,874 | $23,209 | $1,995 | $2,025 | $2,452 | $106,452 | 10 villages |
| Ghana | Volta | NGO CLTS | $8,853 | $1,949 | $26,584 | $555 | $2,888 | $7,501 | $52,175 | 10 villages |
| Ghana | Volta | NGO CLTS + NL training | $9,582 | $54,627 | $29,562 | $2,027 | $3,291 | $18,765 | $126,970 | 10 villages |
| Ghana | Central | NGO CLTS | $977 | $133 | $3,175 | $35 | $167 | $480 | $5,089 | per village |
| Ghana | Central | NGO CLTS + NL training | $1,058 | $4,270 | $3,587 | $169 | $406 | $2,611 | $12,305 | per village |
| Ghana | Upper West | NGO CLTS | $931 | $93 | $1,827 | $37 | $145 | $128 | $3,380 | per village |
| Ghana | Upper West | NGO CLTS + NL training | $1,004 | $6,287 | $2,321 | $200 | $203 | $245 | $10,645 | per village |
| Ghana | Volta | NGO CLTS | $885 | $195 | $2,658 | $55 | $289 | $750 | $5,218 | per village |
| Ghana | Volta | NGO CLTS + NL training | $958 | $5,463 | $2,956 | $203 | $329 | $1,876 | $12,697 | per village |
| Ghana | All | NGO CLTS | $26,958 | $4,076 | $73,428 | $1,241 | $5,837 | $13,101 | $131,773 | 29 villages |
| Ghana | All | NGO CLTS + NL training | $29,145 | $155,928 | $85,052 | $5,545 | $8,971 | $44,718 | $344,169 | 29 villages |
| Ghana | All | Both | $56,103 | $160,004 | $158,480 | $6,786 | $14,808 | $57,819 | $475,942 | 58 villages |
| Ghana | All | NGO CLTS | $930 | $141 | $2,532 | $43 | $201 | $452 | $4,544 | per village |
| Ghana | All | NGO CLTS + NL training | $1,005 | $5,377 | $2,933 | $191 | $309 | $1,542 | $11,868 | per village |
| Ghana | All | Both | $967 | $2,759 | $2,732 | $117 | $255 | $997 | $8,206 | per village |
| Ghana | All | NGO CLTS | $7.83 | $1.18 | $21.33 | $0.36 | $1.70 | $3.81 | $38.27 | per household |
| Ghana | All | NGO CLTS + NL training | $8.80 | $47.08 | $25.68 | $1.67 | $2.71 | $13.50 | $103.92 | per household |
| Ghana | All | Both | $8.31 | $23.69 | $23.46 | $1.00 | $2.19 | $8.56 | $70.46 | per household |
| Ethiopia | Oromia | HEW CLTS | $4,345 | $9,979 | $2,820 | $1,084 | $1,435 | $911 | $20,573 | 1 kebele |
| Ethiopia | Oromia | Teacher CLTS | $7,434 | $17,279 | $1,118 | $1,885 | $1,886 | $633 | $30,235 | 2 kebele |
| Ethiopia | SNNP | HEW CLTS | $4,345 | $7,638 | $2,071 | $842 | $1,110 | $159 | $16,166 | 1 kebele |
| Ethiopia | SNNP | Teacher CLTS | $7,434 | $15,951 | $5,107 | $1,974 | $2,265 | $380 | $33,110 | 2 kebeles |
| Ethiopia | All | HEW CLTS | $8,690 | $17,617 | $4,891 | $1,926 | $2,546 | $1,070 | $36,739 | 2 control kebeles |
| Ethiopia | All | Teacher CLTS | $14,867 | $33,229 | $6,225 | $3,859 | $4,151 | $1,013 | $63,345 | 4 pilot kebeles |
| Ethiopia | All | Both | $23,557 | $50,847 | $11,116 | $5,785 | $6,697 | $2,083 | $100,084 | 6 kebeles |
| Ethiopia | All | HEW CLTS | $4,345 | $8,809 | $2,445 | $963 | $1,273 | $535 | $18,369 | per kebele |
| Ethiopia | All | Teacher CLTS | $3,717 | $8,307 | $1,556 | $965 | $1,038 | $253 | $15,836 | per kebele |
| Ethiopia | All | Both | $3,926 | $8,474 | $1,853 | $964 | $1,116 | $347 | $16,681 | per kebele |
| Ethiopia | All | HEW CLTS | $5.35 | $10.85 | $3.01 | $1.19 | $1.57 | $0.66 | $22.62 | per household |
| Ethiopia | All | Teacher CLTS | $3.87 | $8.66 | $1.62 | $1.01 | $1.08 | $0.26 | $16.50 | per household |
| Ethiopia | All | Both | $4.31 | $9.31 | $2.04 | $1.06 | $1.23 | $0.38 | $18.32 | per household |

**Table S7.** Analysis of cost sensitivity to estimated parameters.

| **Parameter and base value** | | **Change assessed** | | **Program costs** | | | | | | **Change in program cost** | | **Local investments** | | | | | | | | **Change in local investments** | | **Notes** | |
| --- | --- | --- | --- | --- | --- | --- | --- | --- | --- | --- | --- | --- | --- | --- | --- | --- | --- | --- | --- | --- | --- | --- | --- |
|  |  |  |  | **Management** | | **Training** | | **Facilitation** | |  |  | **Local actors** | | **Community activity** | | **Hired labor** | | **Hardware** | |  |  |  |  |
| **Ghana** | **Base cost** | |  | | $8.31 | | $23.69 | | $23.46 | |  | | $1.00 | | $2.19 | | $3.25 | | $8.56 | |  | | per household targeted |
|  | Gas efficiency (21.3 mpg for car, 40 mpg for motorcycle) | | +50% | | - | | - | | -$0.04 | | -0.1% | | - | | - | | - | | - | | 0.0% | |  |
|  |  |  | -50% | | - | | $0.01 | | $0.12 | | 0.2% | | - | | - | | - | | - | | 0.0% | |  |
|  | Depreciation (15% for car, 20% for motorcycle) | | +50% | | - | | $0.01 | | $0.18 | | 0.3% | | - | | - | | - | | - | | 0.0% | |  |
|  |  |  | -50% | | - | | -$0.01 | | -$0.18 | | -0.3% | | - | | - | | - | | - | | 0.0% | |  |
|  | Maintenance and tires ($0.06 and 0.01 per mile) | | +50% | | - | | - | | $0.03 | | 0.1% | | - | | - | | - | | - | | 0.0% | |  |
|  |  |  | -50% | | - | | - | | -$0.03 | | -0.1% | | - | | - | | - | | - | | 0.0% | |  |
|  | Annual mileage (10,000 miles for car, 6,000 for motorcycle) | | +50% | | - | | -$0.01 | | -$0.12 | | -0.2% | | - | | - | | - | | - | | 0.0% | |  |
|  |  |  | -50% | | - | | $0.02 | | $0.36 | | 0.7% | | - | | - | | - | | - | | 0.0% | |  |
|  | Average drive speed (30 mph) | | +50% | | - | | $0.01 | | $0.27 | | 0.5% | | - | | - | | - | | - | | 0.0% | |  |
|  |  |  | -50% | | - | | -$0.01 | | -$0.27 | | -0.5% | | - | | - | | - | | - | | 0.0% | |  |
|  | Travel times (1-2 hrs to district, 15-20 min btwn villages) | | +50% | | - | | $0.04 | | $6.86 | | 12.4% | | $0.14 | | - | | - | | - | | 0.6% | |  |
|  |  |  | -50% | | - | | -$0.04 | | -$6.86 | | -12.4% | | -$0.14 | | - | | - | | - | | -0.6% | |  |
|  | Time-cost, government ($3.13 / hour) | | +50% | | - | | $0.29 | | - | | 0.5% | | $0.15 | | - | | - | | - | | 1.0% | |  |
|  |  |  | -50% | | - | | -$0.29 | | - | | -0.5% | | -$0.15 | | - | | - | | - | | -1.0% | |  |
|  | Value-of-time, NLs, community members ($0.19 / hour) | | +50% | | - | | - | | - | | 0.0% | | $0.35 | | $1.10 | | - | | - | | 9.7% | |  |
|  |  |  | -50% | | - | | - | | - | | 0.0% | | -$0.35 | | -$1.10 | | - | | - | | -9.7% | |  |
|  | Community activity when Plan is not present (23.6 hours / village / month) | | +50% | | - | | - | | - | | 0.0% | | $0.04 | | $0.11 | | - | | - | | 1.0% | |  |
|  |  |  | -50% | | - | | - | | - | | 0.0% | | -$0.04 | | -$0.11 | | - | | - | | -1.0% | |  |
| **Ethiopia** | **Base cost** | |  | | $4.31 | | $9.31 | | $2.04 | |  | | $1.06 | | $1.23 | |  | | $0.38 | |  | | per household targeted |
|  | Gas efficiency (13.8 mpg) | | +50% | | - | | -$0.05 | | -$0.06 | | -0.7% | | - | | - | |  | | - | | 0.0% | |  |
|  |  |  | -50% | | - | | $0.14 | | $0.19 | | 2.1% | | - | | - | |  | | - | | 0.0% | |  |
|  | Depreciation (15%) | | +50% | | - | | $0.20 | | $0.27 | | 3.0% | | - | | - | |  | | - | | 0.0% | |  |
|  |  |  | -50% | | - | | -$0.20 | | -$0.27 | | -3.0% | | - | | - | |  | | - | | 0.0% | |  |
|  | Maintenance and tires ($0.06 and 0.01 per mile) | | +50% | | - | | $0.02 | | $0.02 | | 0.3% | | - | | - | |  | | - | | 0.0% | |  |
|  |  |  | -50% | | - | | -$0.02 | | -$0.02 | | -0.3% | | - | | - | |  | | - | | 0.0% | |  |
|  | Annual mileage (10,000 miles) | | +50% | | - | | -$0.14 | | -$0.18 | | -2.0% | | - | | - | |  | | - | | 0.0% | |  |
|  |  |  | -50% | | - | | $0.41 | | $0.53 | | 6.0% | | - | | - | |  | | - | | 0.0% | |  |
|  | Average drive speed (30 mph) | | +50% | | - | | $0.29 | | $0.38 | | 4.3% | | - | | - | |  | | - | | 0.0% | |  |
|  |  |  | -50% | | - | | -$0.29 | | -$0.38 | | -4.3% | | - | | - | |  | | - | | 0.0% | |  |
|  | Travel times (various times) | | +50% | | - | | $0.41 | | $0.44 | | 5.4% | | $0.07 | | - | |  | | - | | 2.6% | |  |
|  |  |  | -50% | | - | | -$0.41 | | -$0.44 | | -5.4% | | -$0.07 | | - | |  | | - | | -2.6% | |  |
|  | Value of time, (community: $0.07/hour, local actors: $0.29 - $0.40 /hour) | | +50% | | - | | - | | - | | 0.0% | | $0.53 | | $0.61 | |  | | - | | 42.9% | |  |
|  |  |  | -50% | | - | | - | | - | | 0.0% | | -$0.53 | | -$0.61 | |  | | - | | -42.9% | |  |
|  | Community activity, when Plan is not present | | +50% | | - | | - | | - | | 0.0% | | - | | $0.05 | |  | | - | | 1.9% | |  |
|  |  |  | -50% | | - | | - | | - | | 0.0% | | - | | -$0.05 | |  | | - | | -1.9% | |  |

**Table S8.** Management checklist.

| **Category** | **Sub-category** | **Activity** | **Time estimate** | **Comment** |
| --- | --- | --- | --- | --- |
| **(coordinator)** | | | | |
| **Implementation management** | **Work planning** | Scheduling trainings and field visits | ____ hours per week |  |
|  |  | Planning and organizing trainings (including writing TORs, contacting participants, planning activities) | ____ hours per week |  |
|  |  | Other workplanning | ____ hours per week |  |
|  | **Procurement and purchasing** | Renting training venues | ____ hours per week; for ____ weeks preceding each training |  |
|  |  | Renting vehicles | ____ hours per week; for ____ weeks preceding each training |  |
|  |  | Issuing per-diems | ____ hours per training session |  |
|  |  | Other procurement and purchasing | ____ hours per week |  |
|  | **Oversight of LNGO** | Meetings | ____ hours per week |  |
|  |  | Communication (email and phone) | ____ hours per week |  |
|  |  | Review of LNGO field activities and reports | ____ hours per week |  |
|  |  | Other LNGO management and oversight | ____ hours per week |  |
|  | **Reporting** | Progress reporting | ____ hours per month |  |
|  |  | Financial reporting | ____ hours per month |  |
|  |  | Meetings with RICCS, DICCS, EHSD, CWSA | ____ hours per month |  |
|  |  | Other reporting | ____ hours per week |  |
| **Non-implementation activities** | **Natural leader training manual development** | Input and review of draft manuals | ____ hours per week; for ____ weeks |  |
|  |  | Other training manual development work | ____ hours per week; for ____ weeks |  |
|  | **Research** | Hosting USA teams (including preparation for visits) | ____ hours per day during visit; ____ days UNC and USNO were visiting |  |
|  |  | Communication with research team (Emailing and Skype) | ____ hours per week |  |
|  |  | Filling checklists and sending them by email | ____ hours per month |  |
|  |  | Collecting and reviewing LNGO-filled checklists | ____ hours per month |  |
|  |  | Support of household surveying | ____ hours per week; for ____ weeks |  |
|  |  | Oversight of surveying (including reporting to UNC) | ____ hours per week; for ____ weeks |  |
|  |  | Reviewing research documents (including surveys, academic papers, situational assessment) | ____ hours per month |  |
|  |  | Other research support | ____ hours per week |  |
|  | **Dissemination** | Conferences (including travel, preparation, and attendance) | ____ total days |  |
|  |  | Webinars | ____ total hours |  |
|  |  | Other dissemination | ____ hours per month |  |
|  | **Anything not part of the Gates project** | Fundraising efforts | ____ hours per month |  |
|  |  | Other trainings (e.g. gender mainstreaming) | ____ days per [month / quarter] (circle one) |  |
|  |  | Other WaSH projects (e.g. PanAfric CLTS grant) | ____ hours per week |  |
|  |  | Any other time spent not on the Gates CLTS grant | ____ hours per week |  |
| **Implementation (field activities)** | **Training** | Time training local actors (incl. travel, excl. preparation) | (This information is collected using other checklists) |  |
|  | **Facilitation** | Time in villages (excl. oversight and auditing of contractors) | (This information is collected using other checklists) |  |
